# Risk factors for venous thromboembolism in a single pediatric intensive care unit in China

**DOI:** 10.1186/s12959-024-00596-6

**Published:** 2024-03-15

**Authors:** Jintuo Zhou, Yanting Zhu, Ying Liu, Hairong Zhan, Peiguang Niu, Huajiao Chen, Jinhua Zhang

**Affiliations:** Department of Pharmacy, Fujian Maternity and Child Health Hospital College of Clinical Medicine for Obstetrics & Gynecology and Pediatrics, Fujian Medical University, #18 Daoshan Road, Fuzhou, China

**Keywords:** Pediatric, Risk factors, Venous thromboembolism

## Abstract

**Background:**

Analyses of extensive, nationally representative databases indicate a rising prevalence of venous thromboembolism (VTE) among critically ill children. However, the majority of studies on childhood VTE have primarily concentrated on Caucasian populations in the United States and European countries. There is a lack of epidemiological studies on VTE in Chinese children.

**Methods:**

We conducted a retrospective cohort study of data from the Pediatric Intensive Care (PIC) database. Data were obtained and extracted by using Structured Query Language (SQL) and the administrative platform pgAdmin4 for PostgreSQL. Bivariate analyses were conducted in which categorical variables were analyzed by a chi-square test and continuous variables were analyzed by a Student’s t-test. Separate multivariable logistic regressions were employed to investigate the associations between VTE and sociodemographic factors as well as clinical factors.

**Results:**

Our study included 12,881 pediatric patients from the PIC database, spanning the years 2010 to 2018. The incidence rate of pediatric VTE was 0.19% (24/12,881). The venous thrombotic locations were deep venous thrombosis extremities (*n* = 18), superior vena cava (*n* = 1), cerebral sinovenous (*n* = 1), and other deep venous thrombosis (*n* = 4). Univariate analysis showed that age, weight, shock, sepsis, cancer and vasopressor receipt were statistically significant risk factors for pediatric VTE (all *p* ≤ 0.05). After multivariable logistic regression analysis, only shock (aOR: 6.77, 95%CI: 1.33–34.73, *p* = 0.019) and admission for sepsis (aOR: 6.09, 95%CI: 1.76–21.09, *p* = 0.004) were statistically significant associated with pediatric VTE.

**Conclusions:**

In conclusion, data obtained from the Pediatric Intensive Care (PIC) database revealed a prevalence of VTE in pediatric patients of 0.19%. The most common location for venous thrombi was deep venous thrombosis (DVT) in the extremities. We identified that shock and sepsis were statistically significant factors associated with pediatric VTE.

**Supplementary Information:**

The online version contains supplementary material available at 10.1186/s12959-024-00596-6.

## Introduction

Venous thromboembolism (VTE), comprising deep venous thrombosis (DVT) and pulmonary embolism (PE), is becoming increasingly recognized among pediatric patients [1]. In the past, VTE was considered to occur mainly in adults, while VTE in childhood was considered a rare event. With the rising prevalence of utilizing diverse infusion catheters in clinical diagnoses and therapies, there has been a gradual increase in the incidence of VTE among critically ill pediatric patients. Meanwhile, with the continuous improvement of medical imaging, diagnostic equipment and technology, the diagnostic accuracy of VTE in children has been significantly improved. The first epidemiological study on VTE in children was conducted in the 1990s and showed an incidence of VTE of 0.7 per 10,000 children in Canada [2]. Subsequently, annual pediatric VTE rates of 0.14 per 10,000 were reported in the Netherlands and 0.07 per 10,000 in the United Kingdom [3, 4]. In 2000, pediatric VTE was reported to occur in about 20 per 10,000 admissions in North American hospitals, and a study conducted specifically in children’s hospitals revealed a progressive increase in pediatric VTE incidence over time [5, 6]. During the period of 2001 to 2007, the annual incidence of VTE in children in the United States increased by 70%, from 34 to 58 cases per 10,000 [7]. Nevertheless, the majority of studies on pediatric VTE have focused primarily on Caucasian populations in the United States and European countries, and although three decades have elapsed since these pioneering studies, the epidemiology of VTE in Chinese children is still poorly studied.

Thrombotic disease predominantly emerges in children when multiple concurrent risk factors are present. Epidemiological studies have identified several pediatric clinical conditions that may be significantly associated with VTE, including central venous catheter (CVC), congenital heart disease (CHD), cancers, infections, intensive care unit (ICU) stays, mechanical ventilation, surgery, sepsis, total parenteral nutrition (TPN), and vasopressor administration [2, 3, 8, 9]. Overall, our current understanding of VTE risk factors in children is limited to a few reviews originating from single institutions or multicenter national registries within a specific country and it is noteworthy that the incidence and risk factors of VTE in children may differ among different ethnic groups and nationalities [10, 11]. Various risk assessment scales, such as the Wells score, Caprini scale and Padua scale, are currently used to evaluate thrombosis risk in patients [12–14]. However, these scales take into account factors such as pregnancy, advanced age, and oral contraceptives and are not suitable for children. Therefore, there is a requirement for updated data and a comprehensive approach to examining risk factors in a larger patient population to facilitate the formulation of prevention protocols.

The main purpose of our study was to investigate the occurrence and risk factors of VTE in pediatric patients using a large database of children’s hospital in China, aiming to determine the current prevalence of VTE in children, and to explore the associations between socio-demographic and clinical factors with VTE in children.

## Methods

### Study design and data

We conducted a retrospective cohort analysis of data from the Pediatric Intensive Care (PIC) database (http://pic.nbscn.org), which is a large, single-centered, bilingual database that contains information about children admitted to critical care units at the Children’s Hospital of Zhejiang University School of Medicine from 2010 to 2018 [15]. Data for the PIC database were extracted and downloaded from various information systems within the hospital, comprising the hospital’s electronic medical record system, laboratory information system, computerized physician order entry system, nursing information system, anesthesia information management system, and reporting system of various examination departments (such as radiology, ultrasound, ECG, pathology, and others). Following the schema of the MIMIC-III database, structured clinical data encompassing patient demographics, medications, fluid balances, comprehensive laboratory results, and microbiological information (including tests conducted and sensitivities) throughout the patients’ entire hospitalization were collated from various systems. Permission to use the data for this study was obtained from the data extraction and processing staff under the authentication number 12739887.

### Study sample

The PIC database includes information on 13,499 intensive care unit admissions for 12,881 children aged 0 to 18 years. All pediatric patients in the PIC database were included, and for patients with multiple admissions, only the initial admission was taken into account given the difficulty in attributing results to events subsequent hospitalizations. VTE was identified using diagnosis codes based on ICD-10 Clinical Modification (ICD-10-CM) diagnosis codes. The specifics of the ICD-10 diagnosis codes employed for the identification of VTE are outlined in Supplementary Table 1.

### Data extraction

We obtained and extracted all of the data using Structured Query Language (SQL) and the administrative platform pgAdmin4 for PostgreSQL. Demographic data such as ethnicity, age, gender, weight, length of hospital stay, and previously identified risk factors for thrombosis, such as shock, sepsis, cancer, surgical history, use of total parenteral nutrition (TPN), congenital heart disease, and use of vasoactive drugs were collected [2, 3, 8, 9].

### Statistical analysis

The demographic information and potential risk factors of patients with thrombosis were compared with those without thrombosis using univariate and multivariate analyses. The variance inflation factor (VIF) method was employed to assess collinearity among variables. The result revealed no significant collinearity (VIF < 5) among the variables in our analysis. The distribution of the independent variables was examined through Shapiro–Wilk test. Bivariate analyses were then conducted utilizing a chi-square test for categorical variables and a Student’s t-tests for continuous variables. Separate multivariable logistic regressions were employed to investigate the associations between VTE and sociodemographic factors as well as clinical factors. The association between risk factors and thrombosis was assessed using the odds ratio (OR). Risk factors with a *p*-value < 0.1 were incorporated and adjusted for in the multiple logistic regression model. Statistical significance was defined at an alpha level of 5% (*p*-value < 0.05). All statistical analyses were performed using R, version 4.3.2.

## Results

### Participant characteristics

Our study included all pediatric patients from the PIC database. The population included 7,366 (57.2%) male and 5,515 (42.8%) female pediatric patients. The mean age of the included pediatric patients was 2.5 years (Q1–Q3: 0.1–3.4), and the mean hospital stay was 17.4 days (Q1–Q3: 7–20.1). The majority of the pediatric patients were Han ethnicity (98.8%).

### Prevalence and location of VTE

The incidence rate of pediatric VTE was 0.19% (24/12,881 or 19 cases per 10,000 pediatric patients). Locations of thrombi included deep veins of an extremity (*n* = 18), the superior vena cava (*n* = 1), a cerebral venous sinus (*n* = 1), and other DVT that lacks site-specific information (labeled as “Embolism and thrombosis of other specified veins”) (*n* = 4).

### Risk factors for VTE

Table 1 shows the parameters of univariate analysis comparing pediatric patients with or without thrombosis. Univariate analysis showed that age, weight, shock, sepsis, cancer and vasopressor receipt were statistically significant risk factors for pediatric VTE (all *p* ≤ 0.05). After multivariable logistic regression analysis, only shock (aOR: 6.77, 95%CI: 1.33–34.73, *p* = 0.019) and admission for sepsis (aOR: 6.09, 95%CI: 1.76–21.09, *p* = 0.004) were significantly associated with pediatric VTE. (Table 2)

**Table 1:** Comparison of demographic characteristics of patients with and without thrombosis

Variables	Entire sample (*N* = 12,881)	Non-thrombosis (*N* = 12,857)	Thrombosis (*N* = 24)	*p* value
Ethnicity				
Buyei ethnic	19 (0.14)	19 (0.1)	0 (0)	
Han ethnic	12,734 (98.9)	12,710 (98.9)	24 (100)	
Hui ethnic	9 (0.06)	9 (0.1)	0 (0)	
Miao ethnic	43 (0.33)	43 (0.3)	0 (0)	
Tujia ethnic	15 (0.11)	15 (0.1)	0 (0)	
Yi ethnic	11 (0.08)	11 (0.1)	0 (0)	
Others	50 (0.38)	50 (0.4)	0 (0)	
**Age (year)**				
0 ∼ 1	7041 (54.7)	7033 (54.7)	8 (33.3)	< 0.01
1 ∼ 11	5119 (39.7)	5108 (39.7)	11 (45.8)	
11 ∼ 18	721 (5.6)	716 (5.6)	5 (20.8)	
**Sex**				
Female	5515 (42.8)	5508 (42.8)	7 (29.2)	0.25
Male	7366 (57.2)	7349 (57.2)	17 (70.8)	
**Weight (kg)**		11.4 ± 11.5	18.0 ± 15.1	0.04
**Length of stay (day)**		17.4 ± 18.9	18.6 ± 12.7	0.64
**Shock**	66 (0.51)	63 (0.5)	3 (12.5)	< 0.01
**CHD**	2131 (16.5)	2128 (16.6)	3 (12.5)	0.79
**Sepsis**	367 (2.8)	362 (2.8)	5 (20.8)	< 0.01
**Cancer**	505 (3.9)	501 (3.9)	4 (16.7)	< 0.01
**Surgery**	6157 (47.8)	6147 (47.8)	10 (41.7)	0.69
**TPN**	1718 (13.3)	1712 (13.3)	6 (25)	0.16
**VR**	3281 (25.5)	3270 (25.4)	11 (45.8)	0.04
**Death**	960 (7.5)	960 (7.5)	0 (0)	0.316

CHD: Congenital heart disease; VR: Vasopressor receipt; TPN: Total parenteral nutrition; Data are presented as mean (SD) or number (%). Chi-square tests were used for categorical variables, while Student’s t-tests were employed for continuous variables

**Table 2:** Analysis of risk factors for thrombosis

Variables	Univariate analysis		Multivariate analysis	
	Odds ratios (95% CI)	*p* value	Adjusted odds ratios (95% CI)	*p* value
Sex	1.82 (0.75–4.39)	0.18	-	-
Age				
0 ∼ 1	0.53 (0.21–1.31)	0.17	-	-
1–11	Reference	-	Reference	-
11 ∼ 18	3.24 (1.12–9.36)	0.03	2.16 (0.28–16.82)	0.46
Weight (per kg)	1.03 (1.01–1.05)	<0.001	1.00 (0.95–1.05)	0.954
Length of stay	1.00 (0.98–1.02)	0.754	-	-
Shock	29.01 (8.44–99.73)	<0.001	6.77 (1.36–33.73)	0.02
CHD	0.72 (0.21–2.42)	0.595	-	-
Sepsis	9.08 (3.37–24.46)	<0.001	6.09 (1.76–21.09)	0.004
Cancer	4.93 (1.68–14.48)	0.004	2.56 (0.76–8.57)	0.13
Surgery	0.78 (0.35–1.76)	0.548	-	-
TPN	2.17 (0.86–5.47)	0.1	1.45 (0.50–4.21)	0.47
VR	2.48 (1.11–5.54)	0.027	-	-

CHD: Congenital heart disease; VR: Vasopressor receipt; TPN: Total parenteral nutrition

## Discussion

The present study systematically assessed the incidence and risk factors of pediatric VTE through the utilization of a large, pediatric-specific, single-center database. Twelve thousand, eight hundred eighty-one pediatric patients from the PIC database over 9 years were included and the VTE incidence rate was 19 cases per 10,000 pediatric patients. This is consistent with the reported incidence of pediatric VTE range from 0.07 to 21.9 per 10,000 hospital admissions, and with the rising prevalence of utilizing diverse infusion catheters and improvement of diagnostic modalities, the incidence of pediatric VTE increases year by year [2, 6, 11, 16]. Not surprisingly, the prevalence of pediatric VTE was much lower than that in adults [17, 18]. Pediatric VTE follows a bimodal distribution, with the most significant proportion of cases occurring in early infancy, which accounts for up to 20% of all instances [19]. A second peak is observed during adolescence, which represents around 50% of all VTE events in children aged 11 to 18 years old [19]. The reported overall incidence of neonatal thrombosis ranges from 20 to 380 cases per 10,000 neonatal intensive care unit (NICU) admissions [20–23]. In this study, a total of 24 children were diagnosed with VTE, of whom 8 were < 1 year old, accounting for 30%, a bit higher than the ∼20% reported in previous studies. However, because a relatively large proportion of the included population was < 1 year old, the overall incidence of VTE is lower than that of people aged 1–11 years (8/7041 vs. 11/5119). Moreover, the incidence of VTE in this age group in our study was significantly lower than the reported reference incidence of 1.35% [24]. This can be explained by differences in medical level, ethnic background, region and other factors. Our study observed a higher incidence of VTE in pediatric patients aged 11–18 years compared to those aged 0–1 and 1–11 years, aligning with previous reports.

VTE has been increasingly recognized in pediatric patients, especially in neonates in the ICU. In terms of the site of the thrombus, portal venous thrombosis exhibited the highest prevalence among neonatal thrombotic events, and umbilical venous catheterization was identified as a main risk factor [21, 25]. A multicenter, matched case-control study conducted in the United States showed that 85.5% of hospital-acquired VTE in critically ill children was limb deep venous thrombosis, which was often associated with central venous catheterization [26]. Consistently, in the present study, we found the most prevalent site of VTE in pediatric patients was DVT in the extremities.

As for risk factors for VTE, our results showed that age, weight, shock, sepsis, cancer and vasopressor receipt were statistically significant risk factors in univariate analysis, while shock and sepsis were statistically significant in multivariable logistic regression analysis. In a large prospective cohort study of 2,305,380 adults who underwent surgical procedures between 2005 and 2012 at 374 United States hospitals of all types, patients with sepsis were three times more likely to have postoperative arterial or venous thrombosis than other patients during all surgical procedures [27]. A 2023 meta-analysis similarly identified infection and sepsis as risk factors for thrombosis in pediatric patients [23]. Shock was also confirmed a risk factor for VTE by previous studies. Manohar et al. [28] reported circulatory shock as the predominant risk factor for catheter-related internal jugular vein thrombosis, and this could be attributed to the potential reduction in the bioavailability and efficacy of subcutaneously administered low molecular weight heparin during shock [29]. Multiple studies have explored the risk factors for VTE, and CVC has been proved the most important risk factor for pediatric VTE [16, 23]. The reported incidence of CVC-associated thrombosis in pediatric patients varied from 1.03 to 9.3% [30–33]. Furthermore, two meta-analyses yielded pooled odds ratios (ORs) for CVC-associated thrombosis: 3.66 (95% CI: 1.78–7.51) and 2.12 (95% CI: 2.00–2.25), respectively [23, 34]. However, as the PIC database does not provide relevant information regarding the use of CVC in pediatric patients, we were unable to assess the frequency of CVC-associated thrombosis in pediatric patients in our study.

VTE in children significantly increases clinical morbidity and mortality [35, 36]. In our study, we analyzed the association between VTE and mortality in children and, probably due to the small sample size, no significant association was found (Table 1). Although the reported specific mortality of VTE in children may be low, the relatively high all-cause mortality highlights the severity of the underlying diseases in children with VTE, so it is important to identify risk factors for VTE in hospitalized children.

### Limitation

The limitations of this study stem from the inherent characteristics of the administrative discharge database, which is dependent on coding and billing, occasionally resulting in limited granularity and missing data. With the presence of a CVC being the highest risk for thrombosis in children, as the PIC database does not provide relevant information regarding the use of CVC in pediatric patients, we were unable to assess the frequency of CVC-associated thrombosis in pediatric patients in our study. Employing BMI for evaluating the risk of thrombosis in children is considered more scientifically rigorous. However, due to missing height data for over 30% of pediatric patients in the PIC database, we were unable to calculate BMI and assess its association with VTE risk in this population. Variables such as the use of anticoagulants or antithrombotic agents, as well as the diagnosis of underlying disorders of hemostasis/thrombosis, are of significant interest to the pediatric critical care community. Nevertheless, obtaining these variables from the PIC database presents challenges. Consequently, we are unable to incorporate them into our analysis. The study is constrained by its dependence on ICD-10 codes for outcome identification. While we capture VTEs that are formally “diagnosed” through ICD-10 coding, the actual prevalence of VTEs, encompassing undiagnosed and uncoded cases, remains unknown and cannot be estimated. Additionally, this study is a single-center investigation, and there are limitations in extrapolating the findings to other hospitals.

### Conclusions

In conclusion, this analysis of diagnosed VTE in a children’s hospital in China revealed a prevalence of 0.19% among pediatric patients. The most common location for venous thrombi was DVT in the extremities. We identified that shock and sepsis were statistically significant factors associated with pediatric VTE.

### Electronic supplementary material

Below is the link to the electronic supplementary material.

Supplementary Material 1

## Data Availability

Data available on request from the authors.

## Abbreviations

VTE: venous thromboembolism
PIC: Pediatric Intensive Care
SQL: Structured Query Language
DVT: deep venous thrombosis
PE: pulmonary embolism
CHD: congenital heart disease
TPN: total parenteral nutrition
OR: odds ratio
PHIS: Pediatric Health Information System
NICU: neonatal intensive care unit
CVCs: central venous catheters
